# How did Covid-19 impact US household foods? an analysis six months in

**DOI:** 10.1371/journal.pone.0256921

**Published:** 2021-09-15

**Authors:** Kuan-Ming Huang, Ana Claudia Sant’Anna, Xiaoli Etienne

**Affiliations:** 1 Gulf Coast Research and Education Center, University of Florida, Wimauma, Florida, United States of America; 2 Division of Resource Economics and Management, West Virginia University, Morgantown, West Virginia, United States of America; 3 Department of Agricultural Economics and Rural Sociology, Moscow, Idaho, United States of America; University of Florida, UNITED STATES

## Abstract

Using a nationwide survey of primary grocery shoppers conducted in August 2020, we examine household food spending when the economy had partially reopened and consumers had different spending opportunities in comparison to when the Covid-19 lockdown began. We estimate the impact of Covid-19 on household spending using interval and Order Probit regressions. Income levels, age, access to grocery stores and farmers markets, household demographic information, along with other independent variables are controlled in the model. Findings show that middle-class households (with income below $50,000, or with income between $50,000 and $99,999) are less likely to increase their grocery expenditures during the pandemic. Households with children or elderlies that usually require higher food quality and nutrition intakes had a higher probability of increasing their spending during Covid-19 than before. Furthermore, consumers’ spending behaviors were also significantly affected by their safe handing levels and the Covid-19 severity and food accessibility in their residences.

## Introduction

The Covid-19 pandemic has caused enormous disruptions to the global economy. At the beginning of the pandemic, concerns about food shortage led to panic buying as consumers stocked up on groceries [1–3]. During Covid-19, the closure and limited access to restaurant dining, as well as the lifestyle changes (e.g., working from home), have led consumers to change their grocery shopping habits [e.g., 1, 4]. Consumers are also facing higher food prices due to issues in the supply chain such as labor shortage [5, 6] and reduced shipments [7].

Amid this backdrop, we estimate how Covid-19 has affected US household grocery spending behavior, in particular fresh produce and local food purchase and factors driving such changes. Previous studies show that sudden exogenous events, including the 2011 Tohoku Earthquake [8], the 9/11 terrorist attacks [9], Gulf Coast Deepwater Oil Spill [10], Fukushima Nuclear Accident [11], and infectious diseases [12], often led to subsequent changes in short-term shopping behavior. Overall, changes in spending patterns differ depending on the types of events, consumer income, information sources, as well as consumer experience and levels of knowledge. Increased food expenditures due to stockpiling and irrational panic buying are frequently observed at the beginning of extreme events. Consumers consider over-purchasing food, cleaning products, and other necessities as an insurance mechanism to mitigate future uncertainty [2, 8, 13]. Meanwhile, decreased food expenditures after a sudden exogenous event may also occur if the event leads to increased contamination risks, higher health concerns, reduced income, or lower availability of food [e.g., 14, 15].

Given the scope and length of the Covid-19 episode, changes in household purchasing behavior may differ significantly from those observed in previous sudden exogenous events. First, Covid-19 is a global outbreak that affected almost every country in the world, fueling uncertainty across all sectors of the economy. Second, the economic lockdown and lifestyle changes have led to the closures of many businesses, causing unemployment problems and loss of income [16]. Third, production and supply chain disruptions led to limited availabilities and increased retail prices of groceries and necessities [5, 13, 17]. In particular, the U.S. Bureau of Labor Statistics reported the August 2020 food at home and vegetable and fruits price indexes increased 3.7% [18] and 3% [19], respectively, compared to January 2020. Lastly, eligible US households and individuals received stimulus payments that increased their unanticipated income and overall expenditures [20, 21].

These issues created both positive (i.e., stockpiling behaviors, changes of lifestyle, and stimulus payments) and negative (i.e., limited availabilities, loss of income, concerns of health risks) impacts on consumer spending. At the beginning of the pandemic, card and grocery spending increased by approximately 50% from February 26 to March 11 due to consumer panic buying behaviors [2, 7]. Baker, Farrokhnia [22] analyzed transaction-level icial data from August 2016 to March 2020 and found that consumers increased spending on retail and food items in the early stage of the pandemic (from late-February to mid-March) and that the grocery spending at the end of March remained 7.5% higher relative to early 2020. They also indicated that the stimulus payment recipients’ first-month spending increased by $0.30 per dollar of the stimulus received [21]. Dong and Zeballos [23] indicated that between food at home expenditure between April and June 2020 was 3% higher than those recorded for the same period in 2019. In spite of the overall reported increased spending, Ahn and Norwood [24] show that compared to 2016 and 2017, three percentage points more households with children were classified as food insecure in May 2020, and Gundersen, Hake [25] estimate that 17 million more Americans were food insecure in 2020 compared to 2019. Globally, Chinese consumers tend to increase their food stock from 3.37 to 7.37 days after the Covid-19 outbreak [26].

In addition to overall grocery spending, fresh produce spending during the pandemic is another issue worthy of investigation. Fresh produce prices are highly sensitive to supply changes, and given the supply disruptions during Covid-19, the price volatility of fresh produce is likely to be high during the pandemic [1]. Meanwhile, layoffs and reduced incomes due to lockdowns and other restrictions in the short term are exacerbating existing food and nutrition insecurity in many contexts, further reducing the affordability of fresh produce for low-income households [24, 25, 27]. Ellison et al.’s, [4] survey conducted in mid-March to late April 2020 shows that households tend to value nutrition less during the pandemic. The Global Alliance for Improved Nutrition [28] notes that lower-income families tend to choose food with lower safety and quality levels and lower nutritional value during the pandemic. A survey study conducted in the later period, between April and June 2020, by Enriquez and Goldstein [29] found that low-income and benefits-eligible families faced increased financial difficulties and food insecurities during the lockdown period. The food safety concerns and nutrition insecurity problems in the initial lockdown period created greater negative impacts on vulnerable individuals such as young children and pregnant women in the household [27, 30].

Farmers markets may offer a solution for food and nutrition insecurity by providing access to fresh produce and local products. In 2018, 72% of counties reported having at least one farmers market, and 32% of counties reported having at least one farmers market accepting SNAP [31]. In 2019, the number of registered farmers markets had reached 8,140, with an average market day sales of $14,547 and daily household visits of 916 per market [32]. Furthermore, 99.6% of farmers markets sold fruits and vegetables and, 84.7% of markets carried locally grown labeled products [32]. Since farmers markets are a popular outlet for consumers to shop fresh produce and local products, their closure or operational restrictions during the pandemic would certainly limit consumers’ choices and disrupt the local food supply chain. In addition, the closures or operating restrictions of farmers markets would create negative impacts on the local economies and rural communities.

This paper examines the changes in food grocery expenditure, fresh produce expenses, the purchasing behavior of locally grown fresh produce during a later stage of the pandemic and explores the potential factors driving such changes. Most of the recent survey studies on Covid-19 were conducted between February and June 2020, a period generally considered the early pandemic phase [e.g., 4, 24, 29]. In contrast, our online survey was conducted at a later stage of Covid-19. Participants answered the survey between August 12 and 18, 2020.

An important factor that may lead to distinguishing changes in purchasing behavior between the early and the later stages of the Covid-19 is the government assistance program. One-time or temporary government stimulus programs such as the $1,200 stimulus check and the $600 per week enhanced unemployment benefits provided short-term financial reliefs to the households and individuals in need. Still, the absence of continuing programs may leave vulnerable populations unprotected after the expirations of these programs [33]. Indeed, previous findings using data before July 2020 [e.g., 27, 30] show that households and families with young children and pregnant and lactating women suffered the most from the negative impacts such as food and nutrition insecurity in the early phase of the pandemic and the aftershocks may last for years. Moreover, the low-income households (defined as household income < 130% of the federal poverty level) were suffering from food hardships from April to June 2020 [26]. Since no additional stimulus programs or bills were passed after the Coronavirus Aid, Relief, and Economic Security (CARES) Act before August 2020 (at the federal level), we expect the households with lower income or vulnerable members to continue suffering from food spending problems and food hardship after the initial phase. Moreover, we expect the percentage of locally fresh produce purchased to decrease due to the operating restrictions of farmers markets.

We contribute to the literature by analyzing household purchasing trends in August 2020 when the economy had partially reopened, and consumers had different spending opportunities but might have already exhausted their extra income from the first round of government financial assistance, in comparison to the early Covid-19 months. The first-round financial assistance program refers to the CARES Act, an over $2 trillion economic relief package that was passed and signed into law on March 27, 2020. Using a nationwide survey on 514 primary grocery shoppers, we record household spending on food grocery and fresh produce in dollar amount, and the percentage of locally grown fresh produce purchased before and six months after Covid-19 was declared as a pandemic (i.e., August 2020). Shopping behaviors are categorized into three groups: decreased, unchanged, and increased expenditure. Using ordered Probit models, we find that: 1) compared to higher-income households, households with income below $50,000 and those with income between $50,000 and $99,999 are less likely to increase their overall food grocery and fresh produce spending during the pandemic; 2) household demographics such as age and family composition (with kids and elderly) play an important role in household grocery and fresh produce purchasing behavior; 3) Covid-19 severity level, as measured by the number of cumulative Covid-19 cases per 100 people, and food accessibility, as measured by the number of farmers markets and the number of grocery stores and supercenters per 10,000 population significantly affects consumer grocery expenditure.

Our results show that both low-income and middle-class households suffered from food expenditure problems in the middle stage of the pandemic. The finding implies that the food spending problems that households faced in the early pandemic period identified in previous studies continue. Our results provide support for the issuance of financial assistance and stimulus bills that consider low-income and middle-class households and those with vulnerable members. Furthermore, assisting local farmers in adopting new sales channels may help mitigate the loss of businesses due to the operation restrictions of physical farmers markets during the pandemic.

### Survey questionnaire

An online survey on household grocery purchasing behavior was conducted through Qualtrics from August 12 to August 18, 2020. All questions and procedures were approved by the West Virginia University Institutional Review Board before the survey was distributed. Our study does not include minors. Before starting the survey, a written cover letter was presented to the interested participants. Interested participants were informed that the survey is a part of a research project and all participation is completely voluntary. All data were fully anonymized and the survey participants may discontinue the survey at any time. All the recorded responses were from the participants who chose to continue. Our sample quotas and distribution mirrored the five-year average income, education, and age distributions presented in the US Census Bureau 2018 American Community Survey (see S1 Appendix for the detailed quotas). In total, 514 valid responses from the primary grocery shopper of the household were collected. This number is greater than the ideal sample size suggested by Qualtrics—based on the 2015–2019 average number of US households, 120,756,048, the ideal survey sample size for 95% confidence level, 0.5 standard deviation, and 5% margin of error is 385 (See Qualtrics, “Determining sample size: how to make sure you get the correct sample size.” Available at https://www.qualtrics.com/experience-management/research/determine-sample-size/). We focus on the primary food shopper since they have first-hand knowledge of the food budget and the best understanding of the food expenditures of the household, thereby minimizing potential measurement errors caused by recalling and self-reporting expenditures.

To understand how household grocery expenditure has changed due to Covid-19, we asked primary grocery shoppers to answer the following questions for periods both before Covid-19 and during the Covid-19:

- What was/has been your (or your household’s) typical weekly expenses for food purchased during grocery shopping?
- What was/has been your (or your household’s) typical weekly expenses for fresh vegetables and fruits?
- Of the fresh fruits and vegetables you purchased, approximately what percentage was locally grown?

Following Lusk’s [34] food demand survey, we provide respondents with ranges of dollar amounts and ask them to select the one that best describes their purchasing behavior. Based on the average household grocery spending in 2019 [35], we divide the weekly food grocery expenses into ten categories starting at $0, and then with a $25 increment, up until $201 or more. Based on the 2018 weekly fruit and vegetable expenditure as reported by the U.S. Bureau of Labor Statistics [36], we split weekly fresh produce expenses into twelve categories: $0, $1-$5, $6-$10, …, $46-$50, and $51 and more. Individuals that selected a non-zero category in the fresh produce expenditure question were asked the percentage of fresh produce purchased that was grown locally. A total of six numeric options (0%, 1–20%, 21–40%, 41–60%, 61–80%, and 81% or more) and an opt-out option (“I don’t know”) were given. We include the opt-out option to account for the possibility that respondents may be unaware of the origin of the products they purchased. In total, out of the 514 valid responses, 133 respondents chose the opt-out option in at least one period. In follow-up questions, respondents who indicated there was a change in purchasing behavior were asked why the change occurred. Only a small percentage of respondents responded to these questions.

For each question, respondents were asked to select both pre- and during Covid-19 expenditures. To help better understand the Covid-19 timeline and distinguish the “before” and “during” periods, we provide respondents with information that WHO declared Covid-19 as a pandemic in March 2020 at the beginning of the survey. Since the survey was conducted in August 2020, the question encompassed the change in purchase behavior from before March up to August. The longer time span allowed the respondent to have a more informed perception of Covid-19. In addition, compared to previous studies conducted in the early stage of the pandemic that mainly reflect the panic buying and stockpiling behavior, surveying consumers in the later stage of the pandemic allows us to capture a more long-term average change. S2 Appendix provides the actual questions asked in the survey.

Another concern is that some households may have had a different primary grocery shopper during the pandemic due to the changes of household compositions or the members’ relative risks associated with Covid-19. For instance, younger members moving back to their parents’ houses may have become the primary grocery shoppers due to their relatively lower risk of severe illness from Covid-19. However, survey participants are allowed to opt-out for any reasons that include not feeling comfortable to answer or not having sufficient knowledge to answer. Additionally, respondents did not mention lack of knowledge or change in primary shopper in the open-ended portion of the question asking on the reason why expenditures changed or didn’t change. For these reasons, we believe that even if our sample has some “new” primary grocery shoppers, they should have had a sufficient understanding of their households’ current and past food expenditures.

### Data

The detailed demographic information of the surveyed primary food shoppers and variable descriptions are reported in Table 1. Our key explanatory variable, the annual household income level, was categorized into four levels: less than $50,000, $50,000 to $99,999, $100,000 to $149,000, and $150,000 or more. In our discussion of the results, the “base income group” refers to households with income < $50,000, the “middle-income group” refers to households with income of $50,000-$99,999, and the “higher income groups” refer to households with either income $100,000-$149,000 or > $150,000. Of the 514 responses, respondents in each income level from the lowest to the highest accounted for 41.25%, 30.93%, 14.98%, and 12.84% of the sample, respectively. 27.6% of respondents were Supplemental Nutrition Assistance Program (SNAP) participants. The average household size was 3.14 members. 42.22% and 29.96% of households in the sample have at least one member under 18 and at least one member older than 64, respectively. About two-thirds of the respondents are female, which should not come as a surprise since that 65% of the U.S. households’ primary food shopper tend to be female in 2018 [37]. Furthermore, 47.3% of respondents were employed either full-time, part-time, or self-employed, with the remainder being either unemployed, retired, student, homemaker, or unable to work.

**Table 1:** Demographic information of the survey respondents and descriptions of the variables.

**Variable definitions**	**Obs.**	**%**	**Variable definitions**	**Obs.**	**%**
Household income			Age of primary food shopper		
< $50,000	212	41.25%	18 to 24 = 1	51	9.92%
$50,000-$99,999	159	30.93%	25 to 34 = 2	106	20.62%
$100,000-$149,999	77	14.98%	35 to 44 = 3	122	23.74%
$150,000 or more	66	12.84%	45 to 54 = 4	47	9.14%
SNAP Participant	142	27.63%	55 to 64 = 5	97	18.87%
Education of primary shopper	514		65 or above = 6	91	17.70%
Less than high school	60	11.67%	Employed (full/ part time/ self-employed)	243	47.28%
High school	144	28.02%	With at least one kid under 18	216	42.22%
Some college (no degree)	115	22.37%	With at least one member > 64	154	29.96%
Associate’s degree	48	9.34%	Metropolitan area = 1	437	85.02%
Bachelor’s degree	95	18.48%	Owns garden = 1	215	41.83%
Graduate/prof. degree	52	10.12%	Male = 1	166	32.30%
**Variable definitions**				**Mean**	**Std.**
Self-report health level, min (extremely bad) = 1; max = 5				3.76	0.88
Safe practice level, min (not following any food safety procedures) = 0; max = 15				12.27	3.05
Household family size, min = 1; max = 12				3.14	1.86
# of FM selling fresh produce per 10,000 pop, min = 0; max = 4.8				0.18	0.3
# of grocery stores & supercenters per 10,000 pop, min = 0.7; max = 9.4				2.34	1.47
# of Covid-19 cases per 100 pop, min = 0.05; max = 5.13				1.53	0.82

The respondents were divided into metropolitan and non-metropolitan residents using their zip code information. Based on the USDA classification, areas with Rural-Urban code less or equal to (greater than) 3 are considered metropolitan (non-metropolitan) regions. The majority of the respondents (85%) were living in metropolitan areas. By matching a respondent’s zip code with the USDA Food Access Research Atlas dataset, we constructed household food accessibility and shopping location preference measures. These variables include the number of farmers markets selling fresh produce per 10,000 people in 2018 and, the number of supercenters and grocery stores per 10,000 people in 2016 at the zip code level. As shown in Table 1, the number of farmers markets selling fresh produce and the number of supercenters and grocery stores per 10,000 people averaged 0.18 and 2.34, respectively. Furthermore, approximately 42% of respondents owned a personal garden at the time of the survey.

Several measures are constructed to account for Covid-19 and other health risks to the respondent. First, we measure the Covid-19 severity level by the accumulated Covid-19 cases per 100 people on August 12, 2020 (first day of survey) at the county level, based on the respondent’s zip code, using data from USA Facts (available at https://usafacts.org/visualizations/coronavirus-covid-19-spread-map). The number of cases per 100 people averaged 1.53. Second, respondents were further asked to self-report their health status on a scale of 1 to 5 (1 indicates extremely unhealthy and 5 indicates extremely healthy). Individuals in poor health conditions are in general more likely to get severely ill from Covid-19. In our sample, the average self-reported health status was 3.76 (i.e., between fair and good conditions).

Finally, we asked the respondents about their food handling practices. Although there is no scientific evidence suggesting food handling and consumption increases Covid-19 infection risks, a study conducted from May to July 2020 found that consumers were increasingly concerned about Covid-19 virus transmission from consuming food [38]. Practicing good food handing procedures may be one action that consumers take to lower such concerns. Following the food safety guidance suggested in Dietary Guidelines for Americans 2015–2020 [39], respondents were asked to rate on a scale of 0 to 3 (0 is never/almost never and 3 is always/almost always) on the frequencies of practicing the following food handling procedures: i) washing hands with soap and running water before handling food, ii) sanitizing kitchen surface and cutting boards, iii) using one cutting board for fresh produce and separate one for raw meat, iv) rinsing fresh produce under running water just before cutting, eating, or cooking and v) refrigerating or freezing perishables within two hours of purchase. An index reflecting each respondent’s food safety practice levels was computed by summing up the five scores. The food-handling index averages 12.27, out of a 15-point maximum. As discussed earlier, the index may indirectly, to some degree, reflect the respondent’s level of concern about the pandemic and risk aversion in terms of handling food. This variable is included in the regression to control for individuals’ safety measures and perceptions and, potential effects on their food-related shopping behavioral changes.

**Fig 1:**
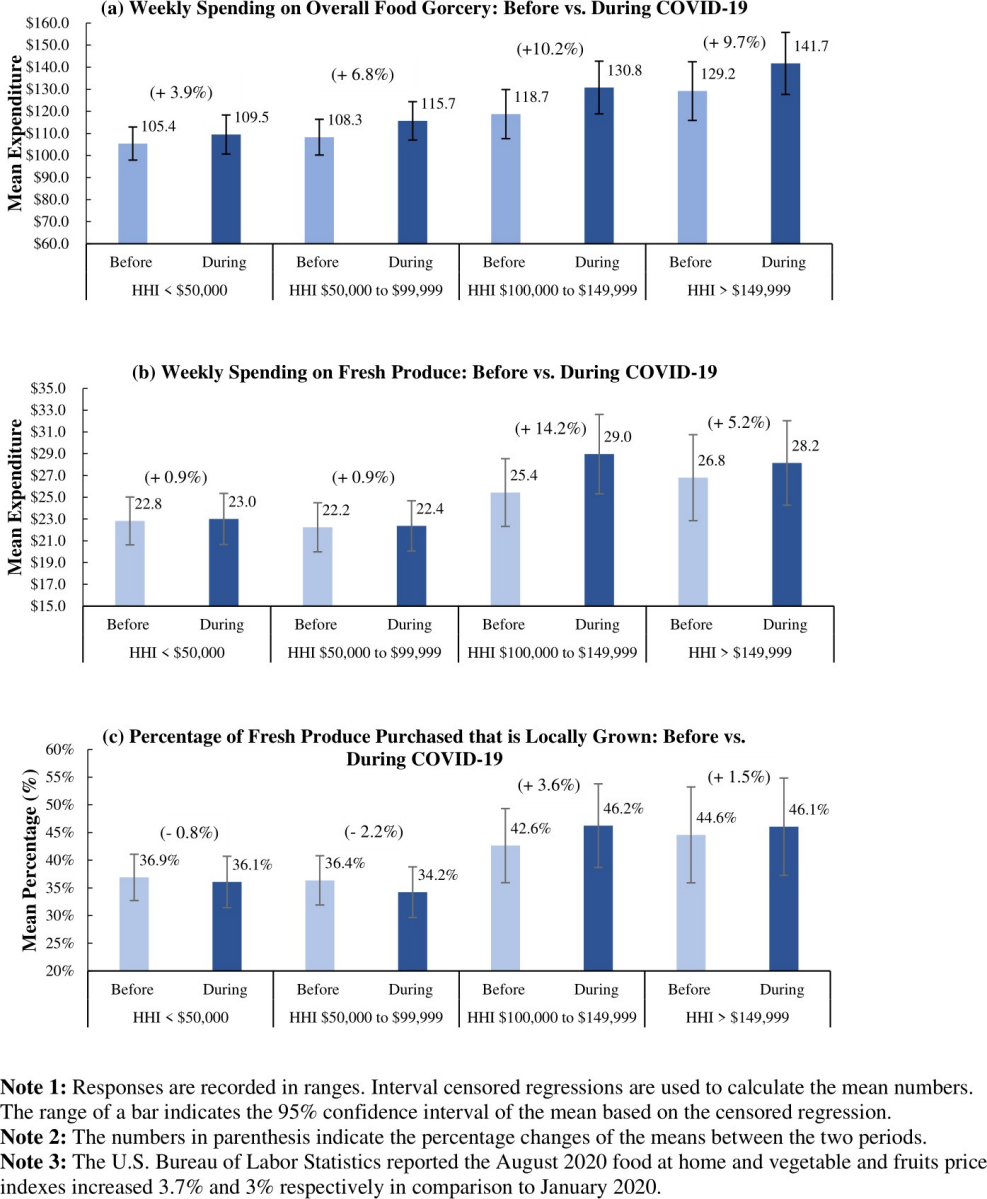
Grocery shopping and fresh produce expenses, and the share of locally grown fresh produce purchased by income level: Before vs. during Covid-19.

We next use interval regressions with only a constant, as in Lusk [34], to estimate average household expenditure levels before and during Covid-19. Fig 1 shows the estimated mean expenditures on food grocery, fresh produce spending, and share of locally grown fresh produce purchased. The mean food grocery expenditure increased across all income levels during Covid-19. The average food grocery spending of the two higher household income (HHI) groups increased 10.2% and 9.7%, respectively, while for the base and middle-income groups, average food grocery expenditures increased by 3.9% and 6.8%, respectively (Fig 1A). These numbers are higher than or close to the food at home price increase from January to August 2020, which ranged around 3.7% as reported by the U.S. Bureau of Labor Statistics [18].

We further find that the average fresh produce spending of the two higher-income groups increased more than 5%, whereas the average fresh produce expenditure of the base and middle-income groups remained almost unchanged (Fig 1B). This result coincides with an earlier study which shows that high-income consumers were willing to pay a higher premium for fresh food reserves during the pandemic in China [26]. Given the price index of fruits and vegetables increased approximately 3% from January to August 2020 [19], this may suggest that the base and middle-income households have had to sacrifice the amount of fresh produce bought to stay within budget. Hence, they might have been sacrificing the nutritional value of the food.

The sample size for the locally grown fresh produce purchasing behavior is smaller than those of the overall and fresh produce expenditures. Respondents could opt out of this question by choosing “I don’t know” if they are unaware of the origin of the fresh produce they purchased. 133 out of 514 respondents opted out of this question. As shown in S1 Appendix, the distribution of the income, age, and education levels of the remaining respondents are still representative and match the quotas and the allocations set in the full sample. Fig 1C shows that higher-income households have on average spent more on local produce than the base and middle-income households during the pandemic—while the average percentage of locally grown fresh produce purchased increased by 3.6% (HHI of $100,000-$149,999) and 1.5% (HHI > $149,999), it decreased by 0.8% in the base income group (< $50,000) and 2.2% in the middle-income group ($50,000 to $99,999).

To construct the dependent variable for the empirical analysis, we compare the surveyed primary food shoppers’ answers with respect to their expenditure level before and during Covid-19 restrictions. We define the household expenditure to have either “increased,” “decreased,” or “remained the same” when a higher, lower, or the same dollar category was selected for the Covid-19 period, respectively. As shown in Table 2, 19.65%, 40.66%, and 39.69% of respondents reported a decrease, no change, and an increase, respectively, in their weekly household spending on food grocery shopping. For fresh produce expenditures, 48.83% of the respondents reported no change to the level of spending during Covid-19, while 20.82% and 30.35% of respondents had decreased and increased their spending, respectively. About half of the respondents (50.39%) purchased the same percentage of locally grown fresh produce during the pandemic as prior to it, and about 25% of the respondents either increased or decreased the portion of locally grown fresh produce in their food expenditure.

**Table 2:** Summary of dependent variable: Changes in spending pattern.

Dependent variables	No. obs	Decrease (value = 1)	Same (value = 2)	Increase (value = 3)	Mean	Std. Dev.
**Weekly spending on food grocery**	514	101 (19.65%)	209 (40.66%)	204 (39.69%)	2.20	0.75
**Weekly spending on fresh produce**	514	107 (20.82%)	251 (48.83%)	156 (30.35%)	2.10	0.71
**Percentage of local fresh produce**	381	94 (24.67%)	192 (50.39%)	95 (24.93%)	2.00	0.71

Notes: The number of observations for local fresh produce is 381 instead of 514 because the respondents who chose the opt-out option “I don’t know” were excluded from the analysis of local fresh produce purchase behaviors.

### Empirical framework and results

To determine what factors drive household food expenditure change during the Covid-19 restrictions, we model the survey data using the ordered Probit model, a method frequently employed in previous studies to analyze ordered categorical data [e.g., 40, 41]. The benefit of using the ordered Probit model is that it takes the latent continuous relationship among the ordinal responses into account, with the results indicating how changes in a predictor affect the probability of observing a particular ordinal outcome [40]. Specifically, the latent continuous dependent variable, y*, is specified in Eq (1) as a linear combination of independent variables,

$$y^{*}=x^{\prime }\;\beta +e,\;e∼NIID[0,1]$$ (1)

where ***x*** is a vector of independent variables, ***β*** is a vector of parameters, and ***e*** is the iid normally distributed error term. The observed ordinal variable, *y*, takes on values 1, 2 and 3 based on the relationship between y* and the thresholds (*μ*_1_, *μ*_2_):

$$y=j↔\mu_{j-1}<y^{*}\leq \mu_{j},\;\text{where}\;j=1,2,3$$ (2)

The probability that a given observation selects alternative *j* can be written as

$$p_{j}=p(y=j)=p(\mu_{j-1}<y^{*}\leq \mu_{j})=F(\mu_{j}-x^{\prime }\;\beta )-F(\mu_{j}-x^{\prime }\;\beta ),$$ (3)

where *F* is the cumulative distribution function of a standard normal distribution. Parameter estimates are obtained by maximizing the log-likelihood function. To account for the possible correlations between the three equations, following the comment of a reviewer of the paper, we also estimate seemingly unrelated regression models using the ordered Probit specification. Parameter estimates are largely consistent with those of the ordered Probit models. Those results are available in S4 Appendix. Greene and Hensher [40] note that computing the marginal effects becomes rather complicated when dealing with multivariate ordered models. They note [40, page 223] that even for bivariate ordered Probit model, partial effects “will be complicated functions of the parameters regardless of how they are defined,” and that “even what margin is of interest is not obvious” given the possible combinations involved. For this reason, we focus our discussions on the single-equation ordered Probit models. The sign of an estimated parameter indicates the direction of the response associated with a change in the independent variable. Since it is difficult to directly interpret the magnitude of the estimated parameters, we compute the marginal effects associated with each independent variable while holding other variables at the sample mean values [40]. For a continuous variable *x*_*r*_, its marginal effect is calculated as $\frac{\partial p_{j}}{\partial x_{r}}=[f(\mu_{j-1}-x\beta )-f(\mu_{j}-x\beta )]\beta_{r}$, where *f* is the probability distribution function. In other words, the marginal effect of *x*_*r*_ is the change in probability of being in outcome *j* due to a change in *x*_*r*_. Since we have three outcomes, there are three marginal effects associated with a given independent variable, i.e., change in probability of increasing, remaining the same, and decreasing level of consumption. The three marginal effects should sum up to zero. For a discrete predictor, its marginal effect can be directly computed as the change in probability of alternative *j* when the independent variable changes from one level to another [40].

The estimation results for each dependent variable, including changes in household overall food grocery expenditure, overall fresh produce expenditure, and the percentage of fresh produce purchased that is grown locally are reported in S3 Appendix. Results suggest that household income and member characteristics are important determinants for both overall food grocery and fresh produce expenses. We further compute the marginal effects of the independent variables for changes in the three ordinal dependent variables, as shown in Table 3. In Table 3, two marginal effects (changes in the probability of increasing consumption and changes in the probability of decreasing consumption) are reported for each independent variable in each regression. Since the two marginal effects are of the opposite sign and sometimes have a similar magnitude for a given independent variable, we focus on the marginal effect for increasing consumption in the following discussion. The marginal effect for changes in the probability of remaining at the same level of consumption can be computed as the negative sum of the other two marginal effects presented. Furthermore, in the following sections, we focus our discussion on variables with significant marginal effects.

**Table 3:** Marginal effects of variables on overall food grocery spending, fresh produce expenditure, and share of locally grown fresh produce purchased.

Variables	Overall food grocery expenditure		Fresh produce expenditure		% of fresh produce locally grown	
	Prob(Decrease)	Prob(Increase)	Prob(Decrease)	Prob(Increase)	Prob(Decrease)	Prob(Increase)
**HHI $50,000-$99,999** ^**a**^	0.002	-0.002	0.005	-0.005	0.049	-0.043
**HHI $100,000-$149,999** ^**a**^	-0.057*	0.083*	-0.096***	0.134***	-0.038	0.042
**HHI >$149,999** ^**a**^	-0.063*	0.094*	-0.062*	0.079*	-0.036	0.039
**SNAP** ^**a**^	-0.031	0.043	0.015	-0.018	0.034	-0.033
**male** ^**a**^	0.057**	-0.08**	0.01	-0.013	-0.019***	0.018***
**household size**	0.005	-0.008	0.003	-0.004	-0.003	0.003
**kid at home** ^**a**^	-0.068***	0.095**	-0.077***	0.094***	-0.008	0.008
**elder at home** ^**a**^	-0.034**	0.047**	0.006	-0.007	-0.071*	0.068*
**employed** ^**a**^	-0.045**	0.063**	-0.01	0.012	0.003	-0.003
**age**	-0.025***	0.035***	-0.026**	0.032**	0.003	-0.003
**education**	-0.007	0.009	0.008	-0.01	-0.008	0.007
**health condition**	-0.015	0.02	-0.029	0.036	0.021	-0.02
**owns garden** ^**a**^	0.043*	-0.059*	0.031	-0.037	0.047***	-0.045***
**live in metro** ^**a**^	-0.024	0.034	0.012	-0.014	-0.021	0.02
**safe handling index**	-0.008***	0.012***	-0.01	0.012*	-0.009**	0.008**
**# farmers market**	-0.736**	1.025**	0.169	-0.206	0.082	-0.079
**# grocery & supercenters**	0.128	-0.179	0.108	-0.131	-0.153**	0.148**
**Covid-19 cases per 100**	-0.036***	0.05***	-0.019***	0.023***	-0.051***	0.049***

***Notes***: ^a^ denotes binary variables. # represents “number of”. One, two, and three asterisks represent statistical significance at 10%, 5%, and 1%, respectively. Parameter estimates for the ordered Probit models are reported in S3 Appendix. The probability of no change, which equals–(probability of increase + probability of decrease) is omitted in the table. For the Household Income categorical variable, the base case is HHI less than $50,000.

Compared to the base income group (< $50,000), households with an annual income exceeding $99,999 and $149,999 are about 8.3 and 9.4 percentage points more likely to increase their food grocery expenditure during Covid-19, respectively. A similar magnitude of the impact is found for households with at least one child. Households with at least one elderly family member are 4.7 percentage points more likely to increase their food grocery spending. Households with an employed primary grocery shopper are also 6.3 percentage points more likely to increase grocery spending. Meanwhile, households with a garden, or whose primary grocery shopper is male, are 5.9 and 8 percentage points less likely to increase spending on food groceries than other households, respectively.

Since age and the safe handling index are discrete ordinal variables, their marginal effects can be interpreted as the percentage points that the household is more/less likely to increase/decrease their food grocery expenditure when the variable of interest increases by one unit. The marginal effects of age indicate that an increase in age level (total of six age groups) of the primary food shopper makes the household 3.5 percentage points more likely to increase overall food grocery spending. Furthermore, when the level of safe handling index increases by one point, the household is 1.2 percentage points more likely to increase its food grocery expenditure. Compared to households living in a region without a farmers market, a primary food shopper residing in an area with 1 farmers market per 1 million population is 1 percentage point more likely to increase his/her food grocery spending. Compared to households living in a county without any Covid-19 cases, a primary food shopper living in a county with 1 Covid-19 case per 100 population is 5 percentage points more likely to increase his/her food grocery expenditure.

For fresh produce expenditure, Table 3 suggests that compared to the base group, households whose incomes fall between $100,000 and $149,999 and those that exceed $150,000 are 13.4 and 7.9 percentage points more likely to increase their expenditures, respectively. A household with kids is 9.4 percentage points more likely to increase their spending on fresh produce compared to those without kids. Furthermore, an increase in the primary grocery shopper’s age level makes the household 3.2 percentage points more likely to increase fresh produce expenditure. Compared to households living in a county without any Covid-19 cases, a primary food shopper living in a county with 1 Covid-19 case per 100 population is 2.3% more likely to increase his/her fresh produce expenditure.

The last two columns of Table 3 display the marginal effects for variables affecting the share of local fresh produce over the total fresh produce purchase. The largest marginal effect comes from the presence of elderly members—households with at least one member older than 64 are 6.8 percentage points more likely to increase the share of locally grown fresh produce purchased. When the level of safe handling index increases by one point, the household is 0.8 percentage points more likely to increase its local fresh produce purchase share. On the other hand, owning a garden makes the household 4.7 percentage points more likely to purchase less, and 4.5 less likely to purchase more locally grown fresh produce. Regarding the accessibility to food outlets, when the number of grocery stores and supercenters per 10,000 population in the neighborhood increases by 1, households are 14.8 percentage points more likely to increase the share of local fresh produce purchase. Lastly, compared to households living in a county without any Covid-19 cases, a primary food shopper living in a county with 1 Covid-19 case per 100 population is 4.9% more likely to increase their local fresh produce share.

## Discussions and conclusions

Existing studies on the impact of Covid-19 on consumers’ food shopping behaviors and insecurity problems primarily focus on the early stage of the pandemic, i.e., March to June 2020 [e.g., 4, 25, 42]. Given the changes in the economic conditions, expirations of government assistance programs, and potential changes in food systems, it is essential to conduct a study focusing on consumers’ shopping behaviors and identifying the vulnerable groups in later stages of the pandemic, when most enhanced benefits packages expired. We contribute to this necessity by surveying household primary shoppers in August about their spending trends during and prior to Covid-19. Using a nationwide survey, we investigate how Covid-19 has affected US household grocery spending behavior, in particular fresh produce and local food purchase and the factors driving such changes. Out of 514 responses, about 40% and 30% of the households reported increased overall food grocery and fresh produce expenditures, respectively, while about 20% of the households decreased their spending on these two categories. About a quarter of the households reduced the share of locally grown fresh produce over the total fresh produce purchase, while more than half of the sample reported no change in local fresh produce shopping behavior.

Interval regression results show that average food grocery spending of the two higher household groups increased by 10.2% and 9.7%, respectively, while for the base and middle-income groups, average food grocery expenditures only increased by 3.9% and 6.8%, respectively (Fig 1). Results from ordered Probit estimations show that higher-income households are between 9 and 9.6 percentage points more likely to increase their spending compared to the base income group (<$50,000). The fact that higher-income groups have a higher probability of increasing their spending compared to lower-income groups should not come as a surprise. The Global Alliance for Improved Nutrition [28] reports that households with lower income faced greater financial constraints, limiting how much they can increase spending to cover inflated food prices. This may force them to compromise for lower food quality/or quantity [28] or substituting canned goods for fresh produce.

Recent studies also show that many households suffered from food hardship and insecurity during the pandemic [25, 29, 42]. Although we are not able to provide direct evidence, our results show that these limitations may also apply to middle-class families. The interval regression results show that average fresh produce spending of the two higher household groups increased 14.2% and 5.2%, respectively, while for both the base and middle-income groups, average food grocery expenditures only increased by less than 1%. We further use the ordered Probit models to show that the two higher-income households are 13.4 and 7.9 percentage points more likely to increase their spending compared to the base income group (i.e., household income less than $50,000), respectively. Indeed, in our follow-up questions on why expenditure on fresh produce decreased, some respondents from the base and middle-income groups indicated that they suffered from income losses and, thus, had to replace fresh produce with less expensive food.

All else equal, households with kids or elderly are significantly more likely to increase their expenditure on food groceries during the pandemic than other households. This could be due to the higher nutrition and quality demand of kids and older adults. Frank and Schvaneveldt [43] reported that households with young children paid more attention to food quality after the Fukushima nuclear accident. In our case, we find that the presence of kids has positively impacted household spending on fresh produce, a highly nutritious type of food during Covid-19. However, having an elderly at home does not significantly affect household fresh produce spending. One possible explanation is that the elderly may take vitamin or fiber supplements to fulfill the needs of these key nutrition supplied by fresh produce, while parents may prefer to offer kids fresh produce over nutrition supplements.

Although having an elderly in the family does not significantly affect fresh produce expenditures, we find that these households are 6.8 percentage points more likely to increase, and 7.1 percentage points less likely to decrease, the share of local fresh produce purchased than other households during the pandemic. This may be due to: 1) the stronger bond older population may have with the local business and food suppliers, preferring to buy from people they know and trusting how they handle food, and; 2) elderly and seniors may have had more difficulty shifting to online shopping [44], preferring to keep buying from local vendors. Although many farmers markets operated with reduced hours and more operating restrictions, the older population may still prefer purchasing local fresh produce. The support of local businesses from households with elderlies during the pandemic is essential for the survival of these operations. Many local businesses, including the local fresh produce suppliers, have faced extremely severe adverse shocks and suffered long-term or permanent loss of business and customers. Our result would suggest that campaigns aiming to promote local and small businesses by providing local/small business maps, statement credits, or coupons may have a greater effect when targeted towards the older population.

Characteristics of the primary household food shoppers such as age, gender, and employment status significantly affect food purchasing behavior during Covid-19. We find that households whose primary grocery shopper is male are more likely to decrease and less likely to increase their food grocery expenses during the pandemic. This is perhaps due to their higher likelihood of ordering take-out meals. The significant positive marginal effects of age on the probability of increasing food grocery and fresh produce expenditures may suggest that primary food shoppers care more about the nutrition content and quality of food as they get older. Older primary food shoppers may also have more exquisite and expensive tastes. This result is consistent with the finding in Hori and Iwamoto [8] that the age of the primary grocery shopper positively affected grocery expenditures among Japanese households after the Tohoku Earthquake. Households whose primary grocery shopper is employed are more likely to increase the overall food grocery expense than those whose primary grocery shopper was unemployed. Employed food shoppers may have fewer financial constraints, allowing them to maintain their food quantity and quality standard at a higher price during the pandemic.

The probability of higher household expenditure on food grocery is positively related to the household’s safe handling index. This indicates that individuals who follow food safety guidance and frequently practice safety procedures are willing to spend more on food quality and/or quantity. Another possible explanation is that consumers with a higher household safe handling index may be more risk-averse. In seeking to minimize exposure to Covid-19, respondents with a higher household safe handling index may have used grocery shopping services (e.g., online shopping and delivery), which may have increased the amount spent on groceries. In a survey conducted by Peña-Lévano, Burney [7], the authors find that consumers have been more likely to purchase food groceries online during the pandemic. These food shoppers also tend to have a higher probability of increasing the share of local fresh produce purchased during the pandemic. They may consider local produce a safer option, presenting lower Covid-19 contamination or food safety risks than the conventional products sourced from other regions. Even if the Covid-19 transmission risk through food is negligible, longer supply chains that involve more people are still subject to more potential sources of Covid-19 infection [45]. Households may also have had more information about the origin and handling of local food and, thus have been willing to purchase more locally grown produce during the pandemic. They may, for instance, personally know the owner of the store and understand their cleaning standards. This result is consistent with the finding of Peña-Lévano, Burney [7] that consumers tend to value food safety and local food attributes more in the long run.

Lastly, we find that households are more likely to increase overall food grocery expenditure, spending on fresh produce, and local fresh produce share during the pandemic in counties with more severe Covid-19 conditions. A possible explanation is that consumers tend to consume more homemade meals and/or be more willing to spend on food quality when the risk of dining out is relatively higher in his/her area. Consumers may also focus more on the nutritional content of food by consuming healthier products, to improve their immune system and lower the risk of contracting Covid-19. Moreover, consumers may believe that the shorter supply chain of local fresh produce reduces Covid-19 contamination risks. Although produce safety may decrease in areas with more severe Covid-19 conditions, the local fresh produce supply chain is still shorter and subject to less contact and transitions than conventional retail fresh produce. For this reason, regardless of the Covid-19 severity level of a consumer’s residency, they might prefer local fresh produce over the conventional one to minimize the exposure to Covid-19 risks.

The preference for local produce may be further highlighted by the higher probability of an increase in food grocery spending when more farmers markets are present in the area, holding everything else constant. This increase in grocery spending could be due to higher spending in either farmers markets or conventional retail stores, or both. Although many of the farmers markets were forced to close temporarily or operate under more restrictions shortly after Covid-19 was declared as a pandemic [6], they soon re-opened with features that minimized Covid-19 related risks. For instance, some farmers markets asked vendors to place an extra table between the cashier and shoppers to ensure 6-feet distancing. Many farmers markets also limited the number of customers to reduce crowding in response to the pandemic.

Our findings provide indirect evidence that the food hardships in the early pandemic stage identified in previous studies [25, 42] may have continued. In addition to the families with no or low income, many middle-class households were also experiencing potential food hardships for the first time [46]. Furthermore, our findings suggest that households with children or elderly members, who tend to require higher food quality and nutrition intakes, have a higher probability of increasing their spending. This may pose a further strain if these households belong in the middle to lower-income categories. Added to this strain is the fact that benefits packages had already expired in mid-August. The potential food insecurity problem among the base and middle-income households may be further exacerbated by the permanent increase in fresh fruit and vegetable prices compared to the pre-pandemic period due to higher production costs, labor shortages, and structural changes in the food industry [5, 7]. Given the significant impact of Covid-19 on food access, policymakers may wish to expedite passing new rounds of stimulus plans to provide additional financial support to households with vulnerable members and facing financial hardship.

On the retail side, the preference for online shopping may create severe financial difficulties for local farmers and businesses who do not have experience setting up online stores or even access to the Internet, adversely affecting the local economy. Some local farmers started agritourism businesses and/or on-site stores that may help offset the financial losses due to restricted farmers market operations. However, not every farm has the resource and knowledge to operate and prompt their own agritourism business. It is essential for the policymakers to develop programs to increase broadband access and to train local farmers on developing alternative sales channels and innovative approaches such as 1) providing farm-to-home delivery; 2) offering online purchasing options, 3) diversifying retail outlets, 4) following social distancing measures at farmers markets.

This study has several limitations. First, we surveyed the consumers’ income but not the wealth and the recent loss of income. Debt and loss of income may significantly affect a consumer’s food shopping behaviors. Since such information was not included in the survey, we are not able to identify how debt and net wealth may have affected our estimation results. Second, we surveyed whether a respondent is a SNAP recipient, but we did not ask for information about his/her eligibility on new Covid-19 food and financial assistance programs provided by federal or local governments. The type of assistance program and the amount of benefit received may affect consumers’ food shopping behaviors differently. Thirdly, we did not survey consumer’ shopping frequency nor shopping location. Such information would help better interpret the results found in the paper. Similarly, detailed information on the items they purchased, the amount of money they spent on each food item, and the detailed food intake before/during the Covid-19 restrictions would help determine whether consumers sacrificed food quality due to financial constraints during the pandemic, as well as whether they suffered from food insecurity. Future studies may wish to collect such information.

Finally, percentage-wise, our survey sample consists less employed respondents (47.3%) than the US national average of 56.5% in August 2020 [47]. Reasons for this lower percentage may include: 1) respondents in between jobs considered themselves as unemployed; 2) about two-thirds of the respondents are females who traditionally have a lower workforce participation rate than men, and working women have been found to hit hard by the Covid-19 pandemic [48]; 3) people with jobs may be less likely to participate in online surveys due to higher opportunity cost of time. Nevertheless, we are confident in our results, since they are, as shown, in line with findings from other studies. Covid-19 impacted households of all income levels and with young and senior family members. The changes in household purchasing expenditures may have had serious implications for financially vulnerable households that require further attention from policymakers.

## Supporting information

S1 Data (XLSX)

S1 Appendix Quota used in the survey. (PDF)

S2 Appendix Survey questionnaries. (PDF)

S3 Appendix Ordered probit estimation results. (PDF)

S4 Appendix Seeminly uncorrelated regression estimation results. (PDF)
