# Supplementary material for: How did Covid-19 impact US household foods? an analysis six months in
Source: PLoS One. 2021 Sep 15;16(9):e0256921. doi: 10.1371/journal.pone.0256921 (PMC8443072; doi:10.1371/journal.pone.0256921)
Supplement: S1 Appendix — (PDF) [file pone.0256921.s002.pdf]

S1 Appendix I: Quota Used in the Survey

	US 5-year average[a] (Quota)	Full sample (n = 514)	Opt-outs dropped (n = 381)
Household income			
< $50,000	42%	41.25%	40.42%
$50,000 to $99,999	30%	30.93%	31.50%
$100,000 to $149,999	15%	14.98%	16.01%
$150,000 or more	13%	12.84%	12.08%
Education level			
Less than high school	12%	11.67%	9.71%
High school	28%	28.02%	28.61%
Some college or Associate's degree	31%	31.71%	31.76%
Bachelor's degree	18%	18.48%	19.42%
Graduate or professional degree	11%	10.12%	10.50%
Age			
18 to 34	30%	30.54%	30.97%
35 to 54	34%	32.88%	37.01%
55 or above	36%	36.57%	32.02%

Notes: [a]: American Community Survey 2014-2018 5-Year Average Estimate
Source: U.S. Census Bureau, 2018
