# Supplementary material for: How did Covid-19 impact US household foods? an analysis six months in
Source: PLoS One. 2021 Sep 15;16(9):e0256921. doi: 10.1371/journal.pone.0256921 (PMC8443072; doi:10.1371/journal.pone.0256921)
Supplement: S2 Appendix — (PDF) [file pone.0256921.s003.pdf]

S2 Appendix II. Survey Questionnaries

What was/has been your (or your household's) typical **weekly** expenses for **food purchased during grocery shopping**?

	0 (0)	$1-$25 (1)	$26-$50 (2)	$51-$75 (3)	$76-$100 (4)	$101-$125 (5)	$126-$150 (6)	$151-$175 (7)	$176-$200 (8)	$201 or more (9)
Before COVID-19 restrictions (C2_before)	○	○	○	○	○	○	○	○	○	○
During COVID-19 restrictions (C2_during)	○	○	○	○	○	○	○	○	○	○

What was/has been your (or your household's) typical **weekly** expenses for **fresh vegetables and fruits?**

	0 (0)	$1-$5 (1)	$6-$10 (2)	$11-$15 (3)	$16-$20 (4)	$21-$25 (5)	$26-$30 (6)	$31-$35 (7)	$36-$40 (8)	$41-$45 (9)	$46-$50 (10)	$51 or more (11)
Before COVID-19 restrictions (C3_before)	○	○	○	○	○	○	○	○	○	○	○	○
During COVID-19 restrictions (C3_during)	○	○	○	○	○	○	○	○	○	○	○	○

Of the **fresh fruits and vegetables** you purchased during the following time periods, approximately what percentage was **locally grown**?

	0 (0)	1-20% (1)	21-40% (2)	41-60% (3)	61-80% (4)	81% or more (5)	I don't know (99)
Before COVID-19 restrictions (C4_before)	○	○	○	○	○	○	○
During COVID-19 restrictions (C4_during)	○	○	○	○	○	○	○
