# Supplementary material for: How did Covid-19 impact US household foods? an analysis six months in
Source: PLoS One. 2021 Sep 15;16(9):e0256921. doi: 10.1371/journal.pone.0256921 (PMC8443072; doi:10.1371/journal.pone.0256921)
Supplement: S3 Appendix — (PDF) [file pone.0256921.s004.pdf]

S3 Appendix III. Ordered Probit Estimation Results

	Overall food grocery		Fresh produce expenditure		% of fresh produce locally	
	**Coefficient**	**Robust S.E.**	**Coefficient**	**Robust S.E.**	**Coefficient**	**Robust S.E.**
HHI $50,000-$99,999[a]	-0.006	0.071	-0.017	0.053	-0.159	0.109
HHI $100,000-$149,999[a]	0.222*	0.122	0.379***	0.107	0.136	0.243
HHI >$149,999[a]	0.251*	0.148	0.230*	0.122	0.127	0.273
SNAP[a]	0.118	0.221	-0.055	0.141	-0.114	0.126
male[a]	-0.217**	0.086	-0.038	0.123	0.062***	0.019
household size	-0.02	0.043	-0.011	0.008	0.012	0.038
kid at home[a]	0.257**	0.102	0.278***	0.061	0.028	0.2
elder at home[a]	0.128**	0.057	-0.02	0.188	0.238*	0.133
employed[a]	0.170**	0.066	0.037	0.087	-0.011	0.082
age	0.095***	0.016	0.094**	0.039	-0.009	0.031
education	0.025	0.059	-0.031	0.045	0.026	0.085
health condition	0.055	0.088	0.106	0.123	-0.071	0.07
owns garden[a]	-0.161	0.099	-0.111	0.129	-0.158***	0.059
live in metro[a]	0.093	0.19	-0.043	0.251	0.069	0.15
safe handling index	0.031***	0.011	0.036*	0.021	0.029*	0.015
# farmers market	2.781**	1.253	-0.611	1.372	-0.275	0.596
# grocery & supercenters	-0.485	0.37	-0.389	0.483	0.515*	0.27
Covid-19 cases per 100	0.135***	0.048	0.069***	0.026	0.171***	0.055
cut 1	0.465*	0.0271	0.300*	0.163	-0.17	0.269
cut 2	1.636***	0.248	1.679***	0.128	1.367***	0.182
Pseudo R-squared	0.034		0.029		0.03	
No. observations	514		514		381	

Notes: [a] denotes binary variables. A joint test (see Greene and Hensher (40)) for the statistical significance of the two cut points was performed for all three models, and testing results show the two cut points are significantly different (p-value: 0.0000) in all models. One, two, and three asterisks represent statistical significance at 10%, 5%, and 1%, respectively.
