# Supplementary material for: How did Covid-19 impact US household foods? an analysis six months in
Source: PLoS One. 2021 Sep 15;16(9):e0256921. doi: 10.1371/journal.pone.0256921 (PMC8443072; doi:10.1371/journal.pone.0256921)
Supplement: S4 Appendix — (PDF) [file pone.0256921.s005.pdf]

S4 Appendix IV. Seeminly Uncorrelated Regression Estimation Results

	Overall food grocery		Fresh produce expenditure		% of fresh produce locally	
	**Coefficient**	**Robust S.E.**	**Coefficient**	**Robust S.E.**	**Coefficient**	**Robust S.E.**
HHI $50,000-$99,999[a]	-0.006	0.154	-0.017	0.153	-0.159	0.180
HHI $100,000-$149,999[a]	0.222	0.222	0.379*	0.220	0.136	0.259
HHI >$149,999[a]	0.251	0.229	0.230	0.226	0.127	0.274
SNAP[a]	0.118	0.132	-0.055	0.131	-0.114	0.151
male[a]	-0.217*	0.117	-0.038	0.117	0.062	0.133
household size	-0.020	0.039	-0.011	0.039	0.012	0.044
kid at home[a]	0.257*	0.144	0.278*	0.142	0.028	0.164
elder at home[a]	0.128	0.144	-0.020	0.142	0.238	0.170
employed[a]	0.170	0.124	0.037	0.122	-0.011	0.142
age	0.095**	0.043	0.094**	0.043	-0.009	0.050
education	0.025	0.052	-0.031	0.051	0.026	0.062
health condition	0.055	0.064	0.106	0.064	-0.071	0.073
owns garden[a]	-0.161	0.109	-0.111	0.108	-0.158	0.125
live in metro[a]	0.093	0.154	-0.043	0.154	0.069	0.178
safe handling index	0.031*	0.017	0.036**	0.017	0.029	0.020
# farmers market	2.781	1.780	-0.611	1.849	-0.275	2.150
# grocery & supercenters	-0.485	0.365	-0.389	0.362	0.515	0.420
Covid-19 cases per 100	0.135**	0.067	0.069	0.066	0.171**	0.077
cut 1	0.465	0.420	0.300	0.417	-0.170	0.478
cut 2	1.636	0.425	1.679	0.422	1.367	0.482

Notes: [a] denotes binary variables. One, two, and three asterisks represent statistical significance at 10%, 5%, and 1%, respectively.
